# Characterization of Protein-Protein Interaction Interfaces from a Single Species

**DOI:** 10.1371/journal.pone.0021053

**Published:** 2011-06-27

**Authors:** David Talavera, David L. Robertson, Simon C. Lovell

**Affiliations:** Faculty of Life Sciences, University of Manchester, Manchester, United Kingdom; Koç University, Turkey

## Abstract

Most proteins attain their biological functions through specific interactions with other proteins. Thus, the study of protein-protein interactions and the interfaces that mediate these interactions is of prime importance for the understanding of biological function. In particular the precise determinants of binding specificity and their contributions to binding energy within protein interfaces are not well understood. In order to better understand these determinants an appropriate description of the interaction surface is needed. Available data from the yeast *Saccharomyces cerevisiae* allow us to focus on a single species and to use all the available structures, correcting for redundancy, instead of using structural representatives. This allows us to control for potentially confounding factors that may affect sequence propensities. We find a significant contribution of main-chain atoms to protein-protein interactions. These include interactions both with other main-chain and side-chain atoms on the interacting chain. We find that the type of interaction depends on both amino acid and secondary structure type involved in the contact. For example, residues in α-helices and large amino acids are the most likely to be involved in interactions through their side-chain atoms. We find an intriguing homogeneity when calculating the average solvation energy of different areas of the protein surface. Unexpectedly, homo- and hetero-complexes have quite similar results for all analyses. Our findings demonstrate that the manner in which protein-protein interactions are formed is determined by the residue type and the secondary structure found in the interface. However the homogeneity of the desolvation energy despite heterogeneity of interface properties suggests a complex relationship between interface composition and binding energy.

## Introduction

Protein-protein interactions (PPIs) underlie biological function at the molecular level. In the yeast *Saccharomyces cerevisiae*, which is the most comprehensively studied organism, the majority of proteins are involved in some sort of complex [1], [2], [3]. Thus, understanding how proteins interact with each other is an important prerequisite for understanding function on a proteome-wide level. However, the exact determinants of specificity, and of change in specificity as the interactome evolves, are poorly understood. To understand fully the energetics of binding, evolution of protein-protein interactions and functional roles of residues in interfaces, a deeper understanding of the interactions interfaces is required.

There have been previous characterisations of several aspects of protein interfaces, for example studying protein-protein [4], [5], [6], [7], [8] and protein-nucleic acid interactions [9]. A number of differences between protein interaction interfaces and the remainder of the protein's surface have been reported[10], [11], [12], [13]. One of the key characteristics that differs is amino-acid composition [4], [5], [14], indicative of different characteristics required for these residues to perform their functional roles.

Knowledge of interface characteristics have been used in a variety of ways, for example the identification of protein interfaces [15], [16]. Of particular interest are determinants of specificity and knowledge of how evolutionary signals in the interface may be used to predict binding specificity[17], [18].

Some broad trends of interface propensities have been identified. Interface patches must be highly accessible, even if most of their individual components are hydrophobic [4]. Thus, interface residues are located in unusual local structural environments [19]. This is particularly important for residues in β-strands, which, when exposed on the protein surface, are likely to be found in interfaces [16]. Long loops are also favoured in interfaces, whereas α-helices are less favoured [20]. In combination, these propensities may contribute to the creation of relatively planar surfaces [4], [21].

PPI interfaces differ between homo-complexes and hetero-complexes. These include differences in amino acid composition, interface size and contact preferences, [6], [7], [22]. Similarly obligate and transient complexes differ in binding characteristics [23]. Obligate interfaces consist mainly of side chain to side chain contacts, whereas the backbone plays a more important role on transient interactions [24]. This will introduce some differences in the ways proteins recognise each other and how they interact.

Interestingly, PPI binding interfaces are heterogeneous, with individual residues making differing contributions to binding and a minority of residues contributing the bulk of the binding energy [25], [26], [27]. Selection pressure also differs within interfaces, giving rise to different patterns of evolutionary conservation [28], [29]. Importantly, the distribution of residues within the interface is not random [8], [20], with differences observed between core (atoms buried upon complex formation) and rim regions (interacting but solvent accessible atoms) [6], [7]. When determining the residue propensities within interfaces it is important to take these differences into account.

Despite a degree of agreement, there are differences between previous studies. Chakrabarti *et al.,* [6] and Bahadur *et al.,* [7] suggested that their binding sites had different amino acid composition and residue propensities when compared with previous studies (*e.g.,* [4], [5]). They suggested that the differences were due to the previous use of a mixture of homo- and heterocomplexes and their distinction between rim and core areas of the interface. All studies also differ in other aspects, including definition of interfaces, calculation procedures and datasets used. Importantly, previous studies use datasets containing complexes from different species. However, evolutionary constraints on protein evolution can arise from a range of sources [30], [31], and these are likely to differ in different species.

The large number of known protein-protein interactions from yeast, and the increase in the size of Protein Data Bank [32] means that we can use interfaces only from *S. cerevisiae*. This limitation to a single species allows us to control for confounding factors associated with selection pressure on residue content, and so give an accurate picture of the relative propensities and roles of specific residue types.

## Methods

### Datasets

Structures of protein complexes were extracted from the PISA (http://www.ebi.ac.uk/msd-srv/prot_int/pistart.html) [33] and PQS (http://www.ebi.ac.uk/pdbe/pqs/index.html) [34] databases. Databases were merged in the following way: for each all-yeast complex, the most likely PISA conformation was retained, excluding monomers, ambiguous and unidentified assemblies; PQS assemblies were kept when there was no representative in PISA. Where several PQS assemblies were available, the one with the most favourable predicted ΔG was chosen. Additionally, some filters were used to ensure the quality and homogeneity of the data: 1) structures containing only alpha carbons were discarded, 2) chains shorter than 50 residues were removed, as many short peptides are synthetic peptides or small protein fragments, and 3) the assigned hydrogen atoms, nucleic acids, ligands and metal ions were removed.

**Figure 1:**
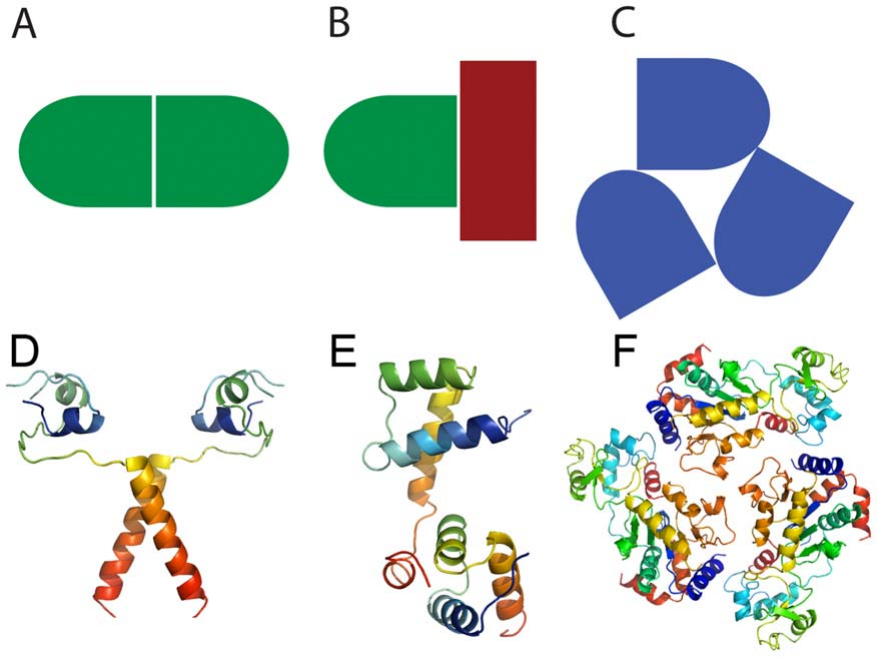
Types of complexes and interfaces. Shapes and colours indicate schematics of protein chains. A a homocomplex with homo-interfaces (both chains use identical surfaces to bind). B a hetero complex with hetero-interfaces (chains are different). C a homocomplex with hetero interfaces (the directly contacting areas are different between interacting chains).

As not all chains in the structural complexes are complete, pairwise global alignments [35] were used to check if complexes were homomeric or heteromeric. Chains were classified as homologous if 1) they were identical, 2) they were 80% similar and retrieve the same top hit from the BLAST-formatted yeast proteome dataset (downloaded from the NCBI at ftp://ftp.ncbi.nih.gov/genomes/Saccharomyces_cerevisiae/), or 3) did not have any hit (neither member of the pair compared) but had more than 80% identical residues. The rest of chains were assigned as being different proteins. We define homocomplexes as those complexes where all the chains were the same and heterocomplexes as those when all the chains were unique. We excluded those multimers with a mixture identical and different chains. In addition, homocomplexes can contain homo- and heterointerfaces depending on the orientation of the chains when interacting (Figure 1). Thus chains binding though identical interfaces at each side have homo-interfaces, otherwise they have hetero-interfaces. In our analyses, we consider only the homointerfaces, as heterointerfaces from homocomplexes are likely to have properties intermediate between the other classes and therefore confuse the analysis. To identify these, pairwise global alignments of the stretches of interaction residues were made, and only those having at least 50% sequence identity were kept. This lower identity threshold was used due to the short length of the aligned sequences. Visual inspection of the data confirmed that they were true homointerfaces. Analysed data is made available as Supporting Information (File S1 contains information on heterocomplexes; File S2 contains information on homocomplexes).

### Definition of interfaces and analyses of the structures

Hydrogen atoms were added using the REDUCE program [36]. PROBE [37] was used to define the interacting atoms. Residues containing at least one interacting atom were classed as interacting. Amino acids that were found not to be directly interacting with other chains were classified as “rim” residues, surface residues or core residues depending on their solvent accessibility in the complex and the disjoint chains. The solvent-accessible area was calculated using NACCESS, which is an implementation of the Lee and Richards algorithm [38]. Rim residues were those losing solvent accessibility but not binding other chains. Thus, they did not contain any interacting atom but did display lower solvent accessibility in the complex than in the disjoint chains. Finally, those residues exposing less than 5% of their area in the disjoint chain were assigned to the core of the protein, whereas the rest were assigned to the surface. Secondary structure was assigned with STRIDE [39].

### Redundancy correction

The most common strategy for doing global analyses of PPIs interfaces includes the selection of complexes representatives or leaving homologues out of the analyses. We believe that this strategy can bias results in three different ways. First, proteins participate in many PPIs. This can be neglected if leaving out homologous interactions. Second, previous research demonstrated that PPIs occur in a number of conformational states. This makes the concept of PPI representative a non-sense. Third, some proteins are analysed in numerous PPIs biasing the background distributions (those calculated using the whole protein surface).

In order to overcome the mentioned biases we used a different approach that consisted in using all the available structures. This permitted to analyse all the available PPIs and binding conformations. As not all proteins were equally distributed, we had to correct for the redundancy of the datasets. We identified two different sources of redundancy: 1) homocomplexes contain two or more identical chains (complex redundancy); 2) some chains are present in more than one structure (dataset redundancy). In order to not overestimate the contribution of any protein, we assigned a contribution for each chain equal to its redundancy factor (RF).


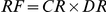
 (1), where CR and DR represent the complex redundancy and dataset redundancy, respectively.


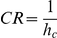
 (2) and 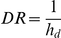 (3), where h_c_ is the number of chains in the homocomplex and h_d_ is the number of structures containing a specific protein at least once. In equations 2 and 3, the analysed protein is included in the count; so, if a protein were unique in the dataset, its contribution is 1, otherwise, it would be less. Obviously, heterocomplexes have a CR equal to 1.

### Calculation of propensities

Propensities (p) show the enrichment or depletion of each feature in the interaction area or the rim compared to the whole of the protein surface.


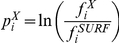
 (4), where 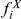 are the frequency of the i^th^ feature in the rim or interaction area, and 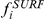 is the frequency of the i^th^ feature in the protein surface.

Frequencies are calculated as the total contribution of the feature in one of the areas compared to the contribution of all present features.


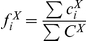
 (5) and 
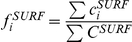
 (6). So, 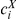
is the contribution of each residue with the i^th^ feature that lies in the interface or rim areas; 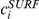
is the contribution of each surface residue with the i^th^ feature; and, 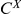 and 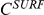 are the contribution of all residues in the interface (or rim) and surface, respectively.

For features based on residue counts (e.g., secondary structure elements), the contribution of each residue is equal to its redundancy factor. Individual amino acid propensities are based on the residue's accessibility (similar to [6], [7]), so the contribution is obtained by multiplying the relative residue surface area by the redundancy factor.


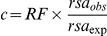
 (7), where RF is the redundancy factor, 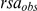 is the solvent-accessible area observed by NACCESS for a particular residue, and 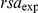 is the expected solvent-accessible area for that residue provided it lied in the middle of an Ala-X-Ala tripeptide.

### Calculation of G_solvation_ using LRT fractional method

The ΔG_solvation_ was calculated by using the fractional solvation method (equation 8) of DT [40]. This method takes into account the different contributions to the solvation energy made by the polar and apolar parts of the amino acids.


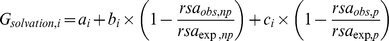
 (8), where a, b and c are constant parameters and derived from linear response theory coupled to molecular dynamics simulations; np and p refer to non-polar and polar parts of the amino acids, respectively.

## Results

### Characteristics of Data Sets

Table 1 shows a summary of the datasets used. We have identified five times as many homo- than heterocomplexes. Heterocomplexes have more chains and more interfaces per complex. However, normalised values, such as the number of interacting residues per interface and the number of interacting atoms per residue are similar.

**Table 1:** Summary of analysed datasets.

	Homocomplexes	Heterocomplexes
Number of PDB structures	449	89
Number of chains	1050 (185)	394 (124)
Chain/Complex	2.34±0.21	4.43±0.79
Number of Interfaces	728 (113)	557 (92)
Interface/Complex	1.62±0.29	6.26±1.69
Number of interacting residues	45242 (6570)	28991 (3815)
Residue/Chain interface	31.07±1.29 (29.13±2.74)	26.02±2.09 (20.74±3.09)
Number of interacting atoms	223169 (32170)	137119 (18762)
Interacting atom/Chain interface	153.28±6.86 (142.61±15.14)	123.09±10.44 (101.96±15.45)
Interacting atom/Residue	4.93±0.03 (4.90±0.08)	4.73±0.04 (4.92±0.10)

Data is presented without any redundancy correction, and with the corrected number between parentheses when relevant. Numbers correspond to number of counts and mean ± standard error (α equals 0.05). Data is per interface; so, residues that are in two interfaces will be counted twice in the number of residues whereas atom/residue will count them separately.

### Individual propensities to be in the rim or interface compared to the whole surface composition

**Figure 2:**
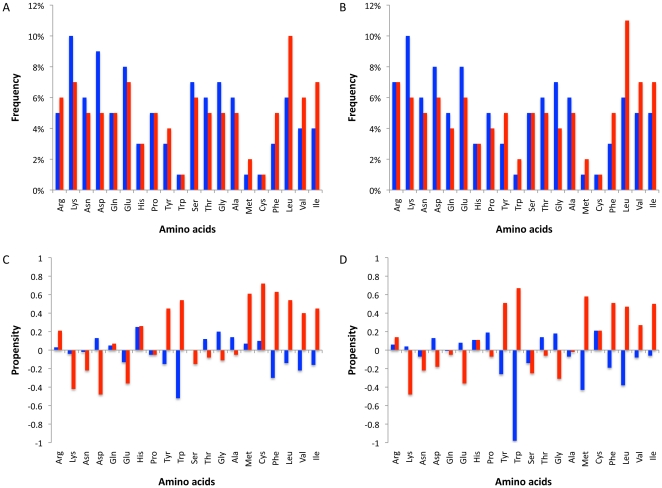
Amino acid composition of interfaces. A. Frequency of residues in homocomplexes. B. Frequency of residues in heterocomplexes. C. Propensities of residues to be in the interface in homocomplexes. D. Propensities of residues to be in the interface in heterocomplexes. Blue bars correspond to the rim area, whereas red bars correspond to the interacting residues. Amino acids are sorted using Kyte and Doolittle table [46], which ranks residues according to their hydropathy. Frequency is based on the number of residues, whereas propensity takes into account the accessibility of each residue in the monomer.

We calculated the frequencies and propensities of interacting residues and those in the “rim” of the interface (Figure 2). The compositional frequencies were based on the number of counts and the individual amino acid propensities based on their accessible area. Similar to previously published work [4], [6], [7], we find that there are large differences between interface and rim residue propensities: propensities within complexes show that rim and interacting residues are inversely correlated (Pearson's r  = −0.39 in homocomplexes; r  = −0.67 in heterocomplexes). We find a smaller enrichment or depletion of residue types than found previously [6], [7]. These differences may be due to the different nature of the data (*e.g.*, one species vs. several species, size of datasets, use of updated datasets) or to different methodologies (*e.g.*, definition of homologous structure/interface, definition of interaction core, redundancy correction). It is likely that the use of our larger data set derived from a single species and the ability for analysing variant interfaces has reduced some differences, at least with respect to the yeast interactome.

As observed previously [4], [6], [7], [10], hydrophobic and aromatic amino acids plus Arg have high propensity to be in the interface. In contrast with previous studies [6], [7] we find that apolar residues are enriched amongst the interacting residues and tend to be just slightly unfavourable in the rim, whereas the aromatic residues are found relatively rarely in the rim despite having some of the highest propensities to interact. This suggests the importance of steric constraints (in addition to the physico-chemical characteristics) in order to establish a favourable interface.

### Comparison of Homo- and Hetero-complex interfaces

There has been previous disagreement about the similarities or differences between the interface composition of homo- and hetero-complexes. Chakrabarti and Janin [6] and Bahadur et al [7] analysed sequence propensities of hetero- and homo-complexes respectively and reported quantitative differences in propensities between the two interface types. The residue propensities of Chakrabarti and Janin [6] differ from those of Jones and Thornton [4] and LoConte et al [5]. Chakrabarti and Janin [6] suggest that the difference is explained by the different partitioning of different types of complexes in previous work.

We find that the frequency of each amino acid is very similar between homo and heterocomplexes in both the rim and interaction areas (Pearson's correlation (r) equal to 0.95 and 0.93, respectively). Moreover, propensities of interacting residues (r = 0.91) are also strongly correlated. However the correlation between hetero-rim propensities and homo-rim propensities is lower (r = 0.71). Together these results suggest that both types homo- and hetero-complexes use amino acids in a similar way to establish interaction contacts, while some small residues (e.g. Gly and Cys) combine with polar amino acids so as to establish a favourable neighbourhood so as to not interfere with the atomic contacts**.**

### Solvation/Desolvation energy

During the binding process, solvent molecules must be removed from the binding interfaces of monomers (*i.e.*, they must be desolvated) so as to establish interactions with their partners. We calculated the solvation energy, which is the amount of energy associated with the return of solvent molecules and the inverse of the desolvation energy.

We find the change in solvation energy on binding is similar between homo- and hetero complexes, whether this is calculated on a “per chain” or “per residue” basis. In addition, the energy is similar between the binding interface (whether rim or core) and the non-interfacial protein surface (see Table 2). The solvation energy is dependent on the types of amino acids present [40]: since there are compositional differences between the interface and the rest of the surface, we may expect that the average solvation energy per residue would differ. The lack of such a difference suggests that there are other factors that compensate for the expected differences in solvation energy. For instance, assuming that not all the interacting residues contribute equally to the binding, we can suppose that the relevance of their solvation, electrostatics and van der Waals energies is not identical. Another possible explanation is that some regions in the non-interacting surface are actually used in binding other proteins that are not in the crystal structure. Thus, many interface regions should have mixed properties: able to be either solvatated or desolvatated.

**Table 2:** G_solvation_ and ΔG_solvation_ per chain and per residue in the different parts of the protein structure.

	Homocomplexes		Heterocomplexes	
	G_solvation_/chain	ΔG_solvation_/chain	G_solvation_/chain	ΔG_solvation_/chain
Core	−120.9±14.8	0.2±0.1	−69.2±18.7	0.2±0.1
Surface	−2576.6±199.8	0.1±0.2	−1891.3±296.6	−0.2±1.0
Rim	−152.0±16.5	18.8±3.0	−147.7±30.5	18.0±4.5
Interaction	−530.9±49.7	235.8±25.2	−464.9±97.6	210.4±51.8
	G_solvation_/residue	ΔG_solvation_/residue	G_solvation_/residue	ΔG_solvation_/residue
Core	−1.7±0.0	0±0.0	−1.6±0.1	0±0.0
Surface	−15.5±0.2	0±0.0	−15.8±0.2	0±0.0
Rim	−16.1±0.7	2.0±0.1	−17.1±0.9	2.1±0.2
Interaction	−15.3±0.3	6.8±0.2	−16.0±0.5	7.2±0.2

Results are mean ± standard error. Units are kcal/mol.

### Atomic interactions between different chains

We next examined the types of atoms that are found in binding interfaces and the interactions they establish. Almost two thirds of interactions are side-chain to side-chain contacts, a larger proportion than previously reported [41].

**Figure 3:**
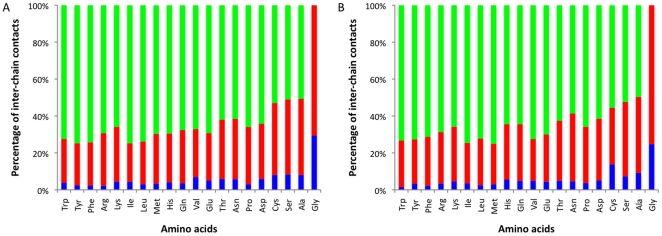
Distribution of inter-chain contacts depending on amino acid type. A. Contacts in homocomplexes. B. Contacts in heterocomplexes. Blue, fraction of main-chain to main-chain contacts. Red, fraction of main-chain to side-chain contact. Green, fraction of side-chain to side-chain contact.1.

We tested whether different residues participate in interactions in similar ways. We find that the type of atoms involved in the interactions may depend on the volume of the residues (see Figure 3). Larger residues tend to have a great number of side chain atoms accessible for specific contacts. Presumably, the larger size will shield the main chain and sterically prevent it from making inter-molecular interactions. Consequently, the greater a residue's volume, the more important it may be for determining the specificity of interactions (Pearson's correlation between the percentage of side-chain contacts and amino acid volume is around 0.75 in both types of complexes). However, there are several interesting exceptions: 1) Asp and Glu are more likely to be involved in interactions through their main chain than Asn and Gln**,** 2) Lys has many contacts involving backbone atoms despite its size, 3) the difference in surface area between Ile and Leu could be the reason for their differences in interaction type despite their similar volume, and 4) Pro has many side-chain interacting atoms despite being a relatively small amino acid. The functional differences between Arg and Lys have been discussed previously [13], and it is thought that the ability for forming H-bonds by the guanidinium and amino groups of Arg and Lys, respectively is the likely cause. Interestingly, there seems to be also some small differences between homo and hetero-complexes for several residues such as Met and Val.

### Secondary structure propensities

**Figure 4:**
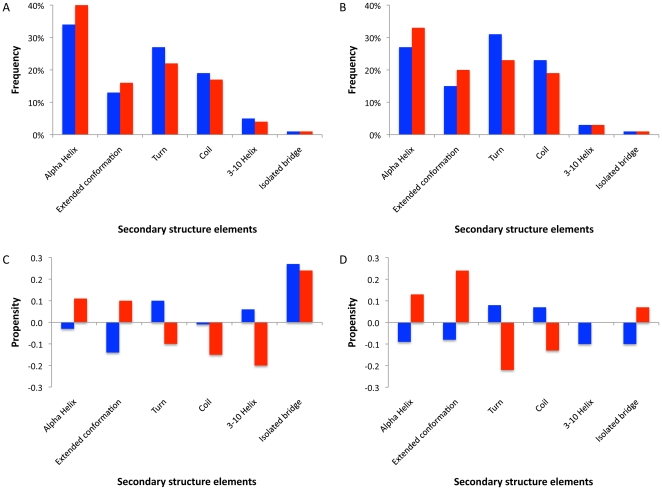
Structural elements in the interfaces. A. Frequency of secondary structure elements in homocomplexes. B. Frequency of secondary structure elements in heterocomplexes. C. Propensities of secondary structure elements to be in the interface in homocomplexes. D. Propensities of secondary structure elements to be in the interface in heterocomplexes. Blue bars correspond to the rim area, whereas red bars correspond to the interacting residues.

In a study of transiently-interacting heterocomplexes, Neuvirth et al [20] found that α-helices are disfavoured within interaction interfaces. Using our larger dataset we calculated the frequencies and propensities for secondary structure elements (Figure 4). The frequencies of each element are highly correlated between homo- and heterocomplexes and between rims and interacting residues (r>0.9 in all cases). This is the case even though the overall secondary structure content of the whole chain varies considerably amongst the proteins in the data set. We find that all types of secondary structure are found within binding interfaces, with α-helices the most common. In both types of complexes, residues within regular secondary structural elements are enriched. Rim regions have little enrichment or depletion for specific types of secondary structure except for a moderate negative propensity for β-strands in homocomplexes.

Types of atomic interactions differ amongst the secondary structure elements (Table 3). Specifically, 1) helices have few backbone-to-backbone contacts, which allow them to establish many specific interactions, 2) the “extended” conformation allows non-specific bonds because it participates in many backbone-to-backbone contacts, and 3) residues outside of secondary structure are difficult to classify because they have many backbone-to-side-chain contacts. Since more than a third of interacting residues are found in α-helices, this points to a major role in specificity recognition. Strands may create stable interaction surfaces that are potentially rich in both specific and non-specific contacts.

**Table 3:** Percentage of atomic contacts per type of structural element.

	Homocomplexes			Heterocomplexes		
	Backbone	Backbone-Side Chain	Side Chains	Backbone	Backbone-Side Chain	Side Chains
Alpha Helix	1.7	26.1	72.2	0.8	25.2	73.9
Extended conformation	11.5	23.8	64.7	9.5	25.8	64.7
Turn	5.8	34	60.2	5.8	35.3	58.9
Coil	5.7	36	58.3	5.4	33.1	61.4
3-10 Helix	5.2	27.2	67.7	2.8	33.7	63.5
Isolated bridge	14.5	34.6	50.9	10.4	35	54.5
PI-helix	NA	NA	NA	0	35.3	64.7

NA states for not available results.

## Discussion

Our analysis of protein interaction interfaces is, to date, based on the largest available dataset, and the study first based on a single species. Our analysis also differs from others in that we take into account that homo-complexes have homo-interfaces and hetero-interfaces. Table 4 shows a summary of our findings and compare them to previous research.

**Table 4:** Summary of main conclusions drawn by this work and comparison with previous research.

Finding	Previous results	Features of alternative dataset
Differences between interface and rim residues	Agreement	28 non-homologous homodimers and 31 heterocomplexes{Jones, 1997 #3}; 70 heterodimers{Bahadur, 2003 #8}; 122 homodimers {Chakrabarti, 2002 #7}
Hydrophobic and aromatic amino acids plus Arg are enriched in the interface	Agreement	28 non-homologous homodimers and 31 heterocomplexes{Jones, 1997 #3}; 70 heterodimers{Bahadur, 2003 #8}; 122 homodimers {Chakrabarti, 2002 #7}
Differences between apolar and aromatic residues in the rim	Disagreement	70 heterodimers{Bahadur, 2003 #8}; 122 homodimers {Chakrabarti, 2002 #7}
No differences between homocomplexes and heterocomplexes	Disagreement	70 heterodimers{Bahadur, 2003 #8}; 122 homodimers {Chakrabarti, 2002 #7}
Similar solvation energy between binding and non-binding areas	Not reported	-
Two thirds of atomic interactions are side-chain to side-chain contacts	Disagreement	356 unique pairs of interacting protein domains {Aloy, 2002 #38}
Important role of α-helices in interfaces	Disagreement	92 unique chains participating in 67 heterodimers {Neuvirth, 2004 #12}

Around two thirds of atomic interactions occurring on yeast interface are between side-chain atoms. If we count the total proportion of side-chain atoms that take part in PPIs (i.e., those in side-chains interactions, and the side-chain portion of mixed interactions) we find that these comprise only 78% of interface atoms, with the remaining 22% of interacting atoms consisting of backbone atoms. Interestingly, these percentages are not evenly distributed among all the amino acid types or the secondary structure elements present in yeast interfaces. Those amino acids with large volumes are more likely to make side-chain interactions than smaller residues, probably because a larger proportion of those residues' atoms are in the side chain. We also find that α-helices are commonly found to make side-chain interactions. Within α-helices the side chains protrude outwards from the axis of the helix, shielding the majority of the main chain atoms from making interactions. At the ends of helices specific side chains often make capping interactions [42], [43], further shielding the main chain. By contrast residues in the edge strands of β-sheets partially expose their main chain atoms [44]. Outside regular secondary structure a range of possibilities are available which may or may not expose main chain atoms.

As previously seen [6], [7], there are differences in amino acid composition between the interacting residues, those residues in the surrounding rim regions and those on the rest of the protein's surface. This should imply differences on solvation/desolvation energy. However, we do not find such differences. It may be that there is selection pressure to maintain surface solvation energy within a relatively narrow range, such that amino-acid substitutions are only accepted if they do not significantly change solvation, regardless of the position on the surface, rim or interface.

In contrast to previously published work [6], [7] we find that there is very little difference between homo-interfaces and hetero-interfaces. This may be due to our larger and species-specific dataset. Alternatively it may be due to the relationship between homo- *vs* hetero-interfaces and obligate *vs* transient interactions. Homo-oligomeric complexes are frequently obligate complexes, *i.e.,* complexes that form soon after folding and remain bound for the lifetime of the complex. Hetero-oligomeric complexes may either be obligate complexes (for example the proteosome) or transient interactions (for example, hormone-receptor complexes). Obligate and transient interactions differ in many of their characteristics [45], and so the previously reported differences between homo- and hetero-interfaces may be due to the conflation of these two factors.

More single-species studies will be possible in the future, making it possible to determine whether divergent species use the same recognition and stabilisation strategies for establishing protein-protein interactions. In the meantime, studies on the evolutionary conservation of the bonds may inform binding specificity. These combined efforts are likely to produce an improvement on the computational methods for predicting protein-protein interactions.

## Supporting Information

File S1 Structural data used from heterocomplexes. Interaction data is presented in three lines. Lines one contains the PDB code, the database we extracted the quaternary structure from and the name of the file (conformation) used. Lines two and three contain the interacting residues and the residues in the rim, respectively. Each residue is identified by the chain and the residue index it has in the original file. Note that quaternary structures databases can contain redundant chain names. Prior to the analyses, chains were renamed to avoid ambiguity; however, the information presented below refers to the chains as they appear in the original files.
(TXT)

File S2 Structural data used from homocomplexes. Interaction data is presented in three lines. Lines one contains the PDB code, the database we extracted the quaternary structure from and the name of the file (conformation) used. Lines two and three contain the interacting residues and the residues in the rim, respectively. Each residue is identified by the chain and the residue index it has in the original file. Note that quaternary structures databases can contain redundant chain names. Prior to the analyses, chains were renamed to avoid ambiguity; however, the information presented below refers to the chains as they appear in the original files.
(TXT)
